# Multiplex PCR Detection of Common Carbapenemase Genes and Identification of Clinically Relevant *Escherichia coli* and *Klebsiella pneumoniae* Complex

**DOI:** 10.3390/antibiotics12010076

**Published:** 2022-12-31

**Authors:** Rujirat Hatrongjit, Peechanika Chopjitt, Parichart Boueroy, Anusak Kerdsin

**Affiliations:** 1 Faculty of Science and Engineering, Kasetsart University Chalermphrakiat Sakon Nakhon Province Campus, Sakon Nakhon 47000, Thailand; 2 Faculty of Public Health, Kasetsart University Chalermphrakiat Sakon Nakhon Province Campus, Sakon Nakhon 47000, Thailand

**Keywords:** PCR, carbapenemase, NDM, KPC, OXA-48-like, *Escherichia coli*, *Klebsiella pneumoniae* complex

## Abstract

Carbapenem-resistant *Enterobacterales* (CRE) species are top priority pathogens according to the World Health Organization. Rapid detection is necessary and useful for their surveillance and control globally. This study developed a multiplex polymerase chain reaction (mPCR) detection of the common carbapenemase genes NDM, KPC, and OXA-48-like, together with identification of *Escherichia coli,* and distinguished a *Klebsiella pneumoniae* complex to be *K. pneumoniae*, *K. quasipneumoniae*, and *K. variicola*. Of 840 target *Enterobacterales* species, 190 *E. coli*, 598 *K. pneumoniae*, 28 *K. quasipneumoniae*, and 23 *K. variicola*. with and without NDM, KPC, or OXA-48-like were correctly detected for their species and carbapenemase genes. In contrast, for the *Enterobacterales* species other than *E. coli* or *K. pneumoniae* complex with carbapenemase genes, the mPCR assay could detect only NDM, KPC, or OXA-48-like. This PCR method should be useful in clinical microbiology laboratories requiring rapid detection of CRE for epidemiological investigation and for tracking the trends of carbapenemase gene dynamics.

## 1. Introduction

The current emergence of carbapenem-resistant *Enterobacterales* (CRE) is an especially concerning antimicrobial resistant (AMR) threat that can result in an important clinical problem associated with resistance to many last-resort antibiotics, making it difficult to treat and leading to high mortality rates and expensive hospital stays [1]. The World Health Organization (WHO) considers the growing AMR issue one of the three major public health challenges of the 21st century, responsible for healthcare costs, long hospitalizations, treatment failures, and death [2]. In addition, the WHO has listed CRE as critical priority pathogens, necessitating the development of new antibiotics against such organisms [3]. The *Enterobacterales*, especially *Escherichia coli*, *Klebsiella pneumoniae*, and *Enterobacter cloacae*, are major causes of hospital-acquired infections that have spread around the globe [1,4].

CRE develops antibiotic resistance through several mechanisms, including through the main mechanism of production of carbapenemases which are enzymes that degrade carbapenem antibiotics [4,5]. They can be divided into CRE with and CRE without carbapenemase production [1,4,5]. Carbapenemase-producing CRE (CP-CRE) are more associated with higher levels of antimicrobial resistance, worse outcomes, and rapid spread via plasmid transmission among bacterial strains, while non-carbapenemase-producing CRE (non-CP-CRE) have been associated with asymptomatic carriage and perhaps less person-to-person transmission [1,6]. Among the carbapenemase types, NDM, OXA-48-like, IMP, VIM, and KPC have been commonly found in different parts of the world [2,4,5]. KPC is the most common carbapenemase in the United States, China, Italy, Greece, and the UK, whereas NDM has now spread worldwide [4,5,7]. VIM and IMP are most prevalent in Southern Europe and Asia, while OXA-48-like is most prevalent in the Mediterranean region, Europe, and Asia, including India, China, Vietnam, and Thailand [4,5,6,7]. Of these carbapenemase types, NDM, KPC, and OXA-48-like have been very frequently detected worldwide [4,5,7]. Furthermore, the major *Enterobacterales* carriers of these carbapenemases are *E. coli* and *K. pneumoniae* [4,5,6].

Surveillance is a crucial element of national prevention and control strategies to control dissemination of CP-CRE. Several countries have systems to monitor acquired carbapenem resistance [8]. For example, diagnostic laboratories in England have a duty to report carbapenemase-producing Gram-negative bacteria isolated from human samples to Public Health England [8]. Therefore, rapid detection and reporting of CP-CRE is one of the top priorities of clinical microbiology laboratories. Recently, there have been increasing numbers of tests available for carbapenemase detection, including colorimetric tests, immunochromatographic assays, matrix-assisted laser desorption ionization-time of flight MS-based tests, phenotypic and molecular methods [9,10]. However, the Clinical and Laboratory Standards Institute (CLSI) recommends the modified carbapenem inactivation method (mCIM), Carba NP, and molecular assay, such as PCR, to determine CP-CRE [11].

PCR is a technique with high accuracy and a rapid turnaround time that is currently inexpensive. Several studies have developed a multiplex PCR (mPCR) to detect the relevant carbapenemase genes [12,13,14,15,16]. However, there is no current PCR approach that detects both carbapenemase genes and *Enterobacterales* species in the same reaction. Herein, we developed an mPCR assay to detect the most prevalent carbapenemase genes, including KPC, NDM, and OXA-48-like, and identify *E. coli*, *K. pneumoniae*, *K. quasipneumoniae*, and *K. variicola* in the same reaction. This mPCR technique could be applied usefully in clinical diagnostic laboratories to determine both common CRE species and the relevant carbapenemase genes at the same time, as well as reducing the turnaround time and saving on costs.

## 2. Results and Discussion

In the current study, an mPCR assay was designed to detect common carbapenemase genes and, frequently, the *Enterobacterales* species carrying those genes. *E. coli* and *K. pneumoniae* complex (*K. pneumoniae*, *K. quasipneumoniae*, *K. variicola*), NDM, OXA-48-like, and KPC, which are common carbapenemase genes disseminated globally, were the target for this mPCR assay. According to several studies, *K. pneumoniae-* and *E. coli*-carrying NDM, KPC, and OXA-48-like, were prevalent in the Netherlands, Spain, the USA, Thailand, China, Korea, Tunisia, Iran, Nepal, and India [6,17,18,19,20,21,22,23,24,25,26,27]. This assay could be applied in these countries, especially in Asia, where there are high prevalence levels of these bacteria carrying the aforementioned carbapenemase genes.

**Figure 1:**
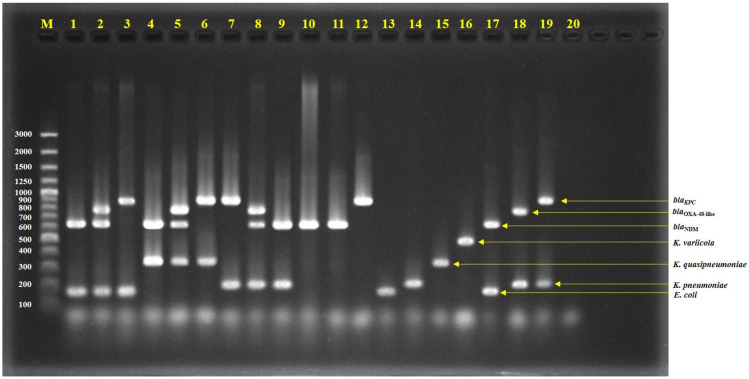
Agarose gel electrophoresis of multiplex PCR-amplified products; lane 1 = Isolate No. C76 (*E. coli* with NDM), lane 2 = Isolate No. C163 (*E. coli* with NDM and OXA-48-like), lane 3 = Isolate No. C1992 (*E. coli* with KPC), lane 4 = Isolate No. C34 (*K. quasipneumoniae* with NDM), lane 5 = Isolate No. C110 (*K. quasipneumoniae* with NDM and OXA-48-like), lane 6 = Isolate No. AMR353 (*K. quasipneumoniae* with KPC), lane 7 = Isolate No. C1985 (*K. pneumoniae* with KPC), lane 8 = Isolate No. C73 (*K. pneumoniae* with NDM and OXA-48-like), lane 9 = Isolate No. C75 (*K. pneumoniae* with NDM), lane 10 = Isolate No. C19 (*E. cloacae* with NDM), lane 11 = Isolate No. C487 (*C. freundii* with NDM), lane 12 = Isolate No. C2135 (*E. asburiae* with KPC), lane 13 = *E. coli* ATCC25922, lane 14 = *K. pneumoniae* ATCC27335, lane 15 = *K. quasipneumoniae* ATCC700603, lane 16 = *K. variicola* ATCC-BAA830, lane 17 = *E. coli* ATCC-BAA2469 (contain NDM), lane 18 = *K. pneumoniae* ATCC-BAA2524 (contain OXA-48), lane 19 = *K. pneumoniae* ATCC-BAA1705 (contain KPC), lane 20 = negative control, and lane M = 100 bp DNA ladder.

We demonstrated the potential utility of the mPCR assay to detect either *E. coli* or *K. pneumoniae* complex with and without carbapenemase genes. The mPCR assay identified the *E. coli* and *K. pneumoniae* complex together with detected NDM, OXA-48-like, and KPC in the same reaction (Figure 1 and Table 1). Our mPCR assay could distinguish the *K. pneumoniae* complex into *K. pneumoniae*, *K. quasipneumoniae*, and *K. variicola* (Figure 1). In the case of *E. coli*, *K. pneumoniae*, *K. quasipneumoniae*, and *K. variicola* without carbapenemase genes, the mPCR assay could identify the species correctly with no carbapenemase genes NDM, OXA-48-like, or KPC (Table 1). In addition, the mPCR assay detected NDM, KPC, or OXA-48-like in *C. fruendii*, *C. werkmanii*, *E. cloacae*, and *E. asburiae*, without bands of *E. coli* or the *K. pneumoniae* complex (Table 1). A limitation of this mPCR assay is that it could not identify *C. freundii*, *E. cloacae*, *E. asburaie*, and *Enterobacterales* species other than the 4 mPCR target organisms. The expansion of this mPCR to detect other *Enterobacterales* species should be the subject of future investigations. In addition, no cross reactivity was observed in other bacteria except the target bacteria and carbapenemase genes.

**Figure 2:**
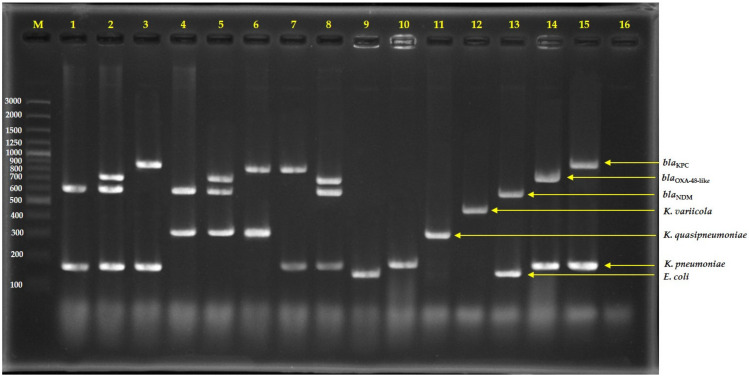
Agarose gel electrophoresis of multiplex PCR-amplified products from direct colonies; lane 1 = Isolate No. C76 (*E. coli* with NDM), lane 2 = Isolate No. C163 (*E. coli* with NDM and OXA-48-like), lane 3 = Isolate No. C1992 (*E. coli* with KPC), lane 4 = Isolate No. C34 (*K. quasipneumoniae* with NDM), lane 5 = Isolate No. C110 (*K. quasipneumoniae* with NDM and OXA-48-like), lane 6 = Isolate No. AMR353 (*K. quasipneumoniae* with KPC), lane 7 = Isolate No. C1985 (*K. pneumoniae* with KPC), lane 8 = Isolate No. C73 (*K. pneumoniae* with NDM and OXA-48-like), lane 9 = *E. coli* ATCC25922, lane 10 = *K. pneumoniae* ATCC27335, lane 11 = *K. quasipneumoniae* ATCC700603, lane 12 = *K. variicola* ATCC-BAA830, lane 13 = *E. coli* ATCC-BAA2469 (contain NDM), lane 14 = *K. pneumoniae* ATCC-BAA2524 (contain OXA-48), lane 15 = *K. pneumoniae* ATCC-BAA1705 (contain KPC), lane 16 = negative control, and lane M = 100 bp DNA ladder.

In addition, we also tried to use the mPCR to directly detect the *E. coli* and *K. pneumoniae* complex together with detected NDM, OXA-48-like, and KPC from pure colonies. As shown in Figure 2, the colony PCR has successfully detected representative strains. This is an alternative method for laboratories because it is not necessary to purify DNA from bacteria growths, and shortens the time and renders unnecessary the equipment required for DNA purification.

The spread of CP-CRE is a global threat to public health. Among CP-CREs, the carbapenemase genes, NDM, OXA-48-like, and KPC, are widely spread globally, as mentioned above. Several phenotypic and genotypic techniques have been applied to detect CRE and CP-CRE [28]. It is vital to diagnose CP-CRE early to undertake the appropriate measures to prevent transmission and to help in implementing effective countermeasures at an appropriate time to initiate early treatment interventions. In addition, the plasmids harboring carbapenemase genes can be transmitted easily among patients. Therefore, a method which can search for genetically diverse CP-CRE isolates would be useful in terms of epidemiological investigation and in tracking the trends of carbapenemase gene dynamics.

PCR is an ideal tool that saves on costs, is rapid, and can identify the type of carbapenemase genes to trace epidemiological dynamics, be proactive in the control and prevention of spread, and provide prompt treatment [12,13,14,15,16]. However, the currently available PCR-detectable carbapenemase genes have not identified the bacterial species [12,13,14,15,16]. Our mPCR assay allows for the rapid testing of common carbapenemase genes (NDM, KPC, OXA-48-like) together with identification of clinical *E. coli* and *K. pneumoniae* complex isolates that frequently harbor these genes; furthermore, the assay does not require prior phenotypical characterization, thus constituting a rapid and valuable tool in the management of infections in hospitals. However, this mPCR has limitations due to the fact that it does not detect IMP genes. The IMP gene is more prevalent in Asia and the South Pacific region than other continents [29]. Therefore, future research directions should include developing this mPCR to detect IMP genes.

## 3. Materials and Methods

### 3.1. Bacterial Strains

As shown in Table 1, this study included 780 *Enterobacterales* strains with known species and carbapenemase genes based on whole-genome sequencing from another study [26,30]: 578 *K. pneumoniae*, 16 *K. quasipneumoniae*, 171 *E. coli*, 6 *Enterobacter cloacae*, 1 *Enterobacter asburiae*, 5 *Citrobacter werkmanii*, and 3 *Citrobacter freundii*.

An additional 159 *Enterobacterales* species without carbapenemase genes including 20 *E. coli*, 20 *K. pneumoniae*, 10 *K. oxytoca*, 23 *K. variicola*, 12 *K. quasipneumoniae*, 20 *K. aerogenes*, 14 *E. cloacae*, 10 *Serratia marcescens*, 20 *Salmonella enterica*, and 10 *C. freundii*, were identified at the species level using conventional biochemical tests; PCR-detected carbapenemase genes described elsewhere were included in the current study [31,32,33]. These bacterial isolates were resistant to either carbapenems or cephalosporins.

We also included reference strains of other bacterial species to evaluate possible non-specific reactions. These strains consisted of: *Achromobacter xylosoxidans* ATCC27061, *Pseudomonas aeruginosa* ATCC9027, *Acinetobacter baumannii* ATCC19606, *Burkholderia cepacia* LMG0122, *Haemophilus influenzae* ATCC10211, *Elizabethkingia meningoseptica* ATCC13253, *Micrococcus luteus* ATCC10240, *Bacillus subtilis* ATCC6633, *Staphylococcus aureus* ATCC700698, *Enterococcus faecalis* ATCC29212, *Streptococcus pneumoniae* ATCC33400, *Leuconostoc lactis* ATCC19256, and *Listeria monocytogenes* ATCC7644. In addition, *E. coli* ATCC-BAA2469 (NDM-1), *K. pneumoniae* ATCC-BAA2524 (OXA-48), *K. pneumoniae* ATCC-BAA1705 (KPC), *K. quasipneumoniae* ATCC700603, and *K. variicola* ATCC-BAA830 were used for PCR reaction control.

The bacterial stains from stock at −80 °C were cultured overnight on sheep blood agar and incubated at 37 °C before performing the DNA extraction.

### 3.2. Primer Design

As shown in Table 2, only primers for NDM and *E. coli* were designed in the current study. The NDM and *E. coli uidA* were retrieved from GenBank under accession numbers NC_023908 and S69414, respectively. These sequences were used as the templates for design of primers by using Primer-BLAST program (http://www.ncbi.nlm.nih.gov/tools/primer-blast/; accessed on 9 December 2022).

### 3.3. Multiplex PCR

Bacterial genomic DNA samples were extracted using ZymoBIOMICS DNA Kits (Zymo Research, CA, USA), following the manufacturer’s instruction. The PCR reaction mixture (25 µL) contained a 1× JumpStart REDTaq ReadyMix (Sigma) and each primer (Table 2). The list of primers used in the mPCR are also presented in Table 2 [34,35,36,37]. The PCR reaction was performed as follows: initial activation of DNA polymerase at 95 °C for 3 min, plus 35 cycles of denaturation at 95 °C for 30 sec, primer annealing and extension at 62.5 °C for 1.30 min, and a final extension at 72 °C for 5 min. A negative control was included in each run, consisting of the same reaction mixture but with water instead of template DNA.

The PCR products (5 µL) were analyzed using gel electrophoresis on 1.5% (*w*/*v*) agarose gel in 0.5 × TBE buffer at a constant voltage of 100 V for 30 min (Mupid exU system; Takara; Tokyo, Japan). The gels were stained with ethidium bromide and visualized under ultraviolet light (GeneGenius Bioimaging System; SynGene; Cambridge, UK). The sizes of the PCR products were determined by comparison with molecular-sized standards (GeneRuler™ 100 bp Plus DNA ladder; Thermo Fisher Scientific; Vilnius, Lithuania).

### 3.4. Colony PCR

Bacteria were cultured on Tryptic Soy Agar for 18 h at 37 °C. A small amount of pure colony was picked up using a toothpick and then suspended in the PCR reaction mixture of each tube. The PCR reaction and gel electrophoresis were done as described above.

## 4. Conclusions

The mPCR assay developed in the current study could detect NDM, KPC, and OXA-48-like together with distinguishing *E. coli*, *K. pneumoniae*, *K. quasipneumoniae*, and *K. variicola* in a single reaction. It should be useful for clinical microbiology laboratories requiring the rapid detection of CRE as one of the critical priority pathogens according to the WHO. In addition, the developed assay should be useful for epidemiological investigation and tracking the trends of carbapenemase gene dynamics.

## Figures and Tables

**Table 1:** *Enterobacterales* species used in this study and mPCR results.

Bacteria	Carbapenemase Gene	N	mPCR Detection						
			*E. coli*	*K. pneumoniae*	*K. quasipneumoniae*	*K. variicola*	NDM	OXA-48-like	KPC
*E. coli* (*n* = 191)	NDM-1	43	+				+		
	NDM-3	1	+				+		
	NDM-4	4	+				+		
	NDM-5	109	+				+		
	NDM-7	1	+				+		
	OXA-48	1	+					+	
	OXA-181	10	+					+	
	KPC-2	1	+						+
	IMP-6	1	+						
	NDM-1 + OXA-181	1	+				+	+	
	none	20	+						
*K. pneumoniae* (*n* = 598)	NDM-1	201		+			+		
	NDM-4	1		+			+		
	NDM-5	5		+			+		
	NDM-9	1		+			+		
	OXA-48	13		+				+	
	OXA-181	147		+				+	
	OXA-232	77		+				+	
	KPC-2	1		+					+
	IMP-14	5		+					
	GES-5	1		+					
	NDM-1 + OXA-181	11		+			+	+	
	NDM-1 + OXA-232	114		+			+	+	
	NDM-1 + GES-5	1		+			+		
	none	20		+					
*K. quasipneumoniae* (*n* = 28)	NDM-1	12			+		+		
	IMP-14	2			+				
	NDM-1 + OXA-181	2			+		+	+	
	none	12			+				
*K. variicola* (*n* = 23)	none	23				+			
*K. oxytoca* (*n* = 10)	none	10							
*K. aerogenes* (*n* = 20)	none	20							
*E. cloacae* (*n* = 20)	NDM-1	5					+		
	OXA-181	1						+	
	none	14							
*E. asburiae* (*n* = 1)	KPC-2	1							+
*C. freundii* (*n* = 13)	NDM-1	3					+		
	none	10							
*C. werkmanii* (*n* = 5)	NDM-1	5					+		
*S. enterica* (*n* = 20)	none	20							
*S. marcescens* (*n* = 10)	none	10							

+ = mPCR positive; blank = mPCR negative.

**Table 2:** Primers used for detection of antibiotic resistance genes.

Primer Name	Sequences (5′—3′)	Final Conc. of Primers (µM)	Species/Gene	PCR Product Size (bp)	Reference
KpnWaaQ-F	CGG ATC CTG GTC ATT AAG CTG	0.5	*K. pneumoniae*	217	[34]
KpnWaaQ-R	ATT GCA TCT TCA GCT GAT ACC TTT	0.5			
KpnCpxLEN-F	CAC GCT GCG YAA ACT ACT GAC YGC GCA GCA	0.5	*K. variicola*	489	[35]
KpnCpxOKP-F	GGC CGG YGA GCG GGG CTC A	0.5	*K. quasipneumoniae*	348	
KpnCpxDeO-R *	AGA AGC ATC CTG CTG TGC G	1.0	*K. quasipneumoniae* and *K. variicola*	-	
ECuidA-F	GGG AAT GGT GAT TAC CGA CGA AAA CGG C	0.1	*E. coli*	175	This study
ECuidA-R	ACA GAC GCG TGG TTA CAG TCT TGC G	0.1			
NDM-F	AAC GGT TTG GCG ATC TGG TTT TC	0.7	*bla*_NDM_	627	This study
NDM-R	GGC GGA ATG GCT CAT CAC GAT C	0.7			
Oxa-48F	TTG GTG GCA TCG ATT ATC GG	0.7	*bla*_OXA-48-like_	733	[36]
Oxa-48R	GAG CAC TTC TTT TGT GAT GGC	0.7			
KPC-F	ATG TCA CTG TAT CGC CGT CT	0.5	*bla*_KPC_	893	[37]
KPC-R	TTT TCA GAG CCT TAC TGC CC	0.5			

* This primer is a reverse primer for KpnCpxOKP-F and KpnCpxLEN-F.

## Data Availability

Not applicable.
